# An analysis of the molecular evolution of Hepatitis B viral genotypes A/B/D using a Bayesian evolutionary method

**DOI:** 10.1186/1743-422X-10-256

**Published:** 2013-08-10

**Authors:** Guangyu Xu, Chengguo Wei, Yuqi Guo, Chao Zhang, Nan Zhang, Guoqing Wang

**Affiliations:** 1 Pharmaceutical College, Beihua University, Jilin city, Jilin 132011, China; 2 Key Laboratory of Zoonosis, Ministry of Education, Norman Bethune College of Medicine, Jilin University, Changchun, Jilin 130021, China; 3 Department of Emergency, The First Affiliated Hospital of Jilin University, Changchun, Jilin 130021, China

**Keywords:** HBV, Genotypes, Bayesian analyses, TMRCA

## Abstract

**Background:**

Hepatitis B virus (HBV) infection is a major global health problem. The infectious virion contains an inner “core particle”, which is made of 180 or 240 copies of core protein, alternatively known as hepatitis B core antigen, or HBcAg which encloses the viral genome.

**Method:**

In this study, we characterized HBV genotypes and used Bayesian analyses to estimate date of emergence of the most recent common ancestor (TMRCA) of three HBV genotypes, A, B, and D.

**Results:**

We estimated that the rate of evolution of HBV core protein gene to be 1.127 (0.925–1.329, 95% HPD) substitutions per site per year. The TMRCA of HBV for genotypes A, B, D were 118 (54–194, 95% HPD) year, 184 (78–323, 95% HPD) year and 133 (65–230, 95% HPD) year, respectively. Demographic histories of the HBcAg gene showed that the relative genetic diversity had a sharp increase within the first 10 years of its emergence.

**Conclusion:**

Using a bayesian evolutionary method to predict the outbreak trends of HBV through evolutionary trees of HBV, and provide theoretical foundations for clinical prevention and treatment of HBV.

## Background

HBV is a genus of DNA viruses which infects the humans causing acute and chronic hepatitis [1]. The World Health Organization estimates that more than 2 billion people have been infected with the hepatitis B virus [2], of which there are 350 million chronic carriers [3]. In recent years, there have been many studies on HBV genotypes and their clinical relationships. It has been shown that HBV genotypes reflect the natural heterogeneity between virus strains more accurately than serotypes.

There are ten different HBV genotypes (A-J) whose prevalences are variably distributed geographically [4]. Genotype A is common in sub-Saharan Africa, Northern Europe and West Africa. Genotypes B and C are highly prevalent in Asia. Genotypes D is highly prevalent in Asia and Africa, and D1 in North Africa, Europe, Central Asia, D2 in North Europe, Russia, Japan (Ehime), D3 in South Africa, Europe, D4 in Australia, D5 in East India [5,6]. Genotype E has been reported in West Africa. Genotype F is found in Central and South America, while genotype G is found in France, Germany and the United States. The eighth genotype, H, has been reported to be present in Central America [7]. At present, genotype I has been described in Vietnam and Laos [8,9]. The newest HBV genotype, J, was identified in the Ryukyu Islands in Japan, and this genotype has a close relationship with gibbon/orangutan genotypes, and human genotype C [10]. There are many applications behind the study of genotypes in HBV. These include elucidation of novel mechanisms of disease pathogenesis, development of biomarkers for disease prognosis or treatment outcome, and identification of potential therapeutic targets. At the same time, there were many people have reported the evolutionary analysis of HBV by different regions of genes and genomes [11,12].

HBV is characterized by high rates of replication (10^12-13^ virions/day), and high rates of mutation (10^10-11^ point mutation/day) which increase the likelihood of the appearance of conserved changes which ultimately can lead to the emergence of new genotypes. The various HBV genotypes are associated with differences in pathogenicity [5], disease progression [13] and responses to antiviral drugs [14].

In this article, we carried out genotype classification of HBV according to the HBcAg (core) gene by the Bayesian method to estimate date of emergence of the most recent common ancestor (TMRCA) of three common HBV genotypes. We predicted the outbreak trends of HBV through evolutionary trees of HBV, and provide theoretical foundations for clinical prevention and treatment of HBV.

## Material and methods

### Sequence collection

A total of 580 HBcAg gene sequences from GenBank were downloaded (http://www.ncbi.nlm.nih.gov), of which 153 had known collection dates, genotype and isolate, and country of origin. Samples obtained between 1990 and 2012 were retrieved for analysis (the accession numbers of these sequences are available through biowcg@yahoo.com). These sequences were processed using BioPerl to generate the format required for manipulation by BEAST 1.6 [15]. Sequences were aligned using MEGA5 software [16], and edited with the SEAL software (available at http://tree.bio.ed.ac.uk/software/seal/). Any missing nucleotides were coded as “missing characters” in the nexus block because BEAST cannot perform the alignments.

### Genotyping analysis

Using the HBV genotype data, the strains were collected using the Markov Chain Monte Carlo method. MCMC together with the information on collection dates, using Bayesian analyses were performed to estimate the time emergence of the most recent common ancestor (TMRCA) [17]. This information led us to make inferences about the oldest, and also the youngest HBV genotypes. The results were summarized using the program TreeAnnotator 1.6.1, and the maximum clade credibility (MCC) tree was constructed. FigTree was used to display the tree.

### Bayesian MCMC evolutionary analyses

After the TMRCA was done with BEAST 1.6, convergence was inspected using Tracer v1.6, with uncertainties addressed as 95% HPD intervals. Analyses were performed using the Hasegawa-Kishino-Yano (HKY) nucleotide substitution models, with a gamma-distributed among-site rate variation with four rate categories [18]. We did the Bayesian MCMC analysis for 50 million states, and sampled every 50,000 states. We set a burn-in of 2 million states for the posterior probabilities, and then showed the results using Tracer, version 1.6. We used Bayesian skyline plots to show the relative viral genetic diversity for HBV core protein gene [19].

### About tMRCA

The most recent common ancestor (MRCA) of any set of organisms is the most recent individual from which all organisms in the group are directly descended. Such time to MRCA (TMRCA) estimates can be given based on DNA test results and established mutation rates as practiced in genetic genealogy, or by reference to a non-genetic, mathematical model or computer simulation [17].

## Results and discussion

### Phylogenetic analysis

**Figure 1:**
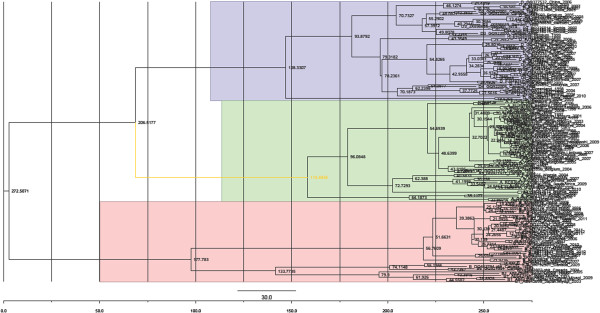
**The maximum clade credibility (MCC) tree was estimated using Bayesian analysis with HBcAg gene sequences about ~660 bp of HBV.** The key nodes above the respective nodes were used to show the posterior probabilities. The green color depict genotype D (n = 54), and blue pink color display genotype A (n = 55) and B (n = 44).

The most common HBV genotype is C, followed by B. However, most of the available data are on genotypes A, B and D. Therefore, we undertook to study the evolution of HBV by analyzing these three genotypes. A total of 580 HBcAg gene sequences were downloaded from GenBank (http://www.ncbi.nlm.nih.gov), of which 153 sequences had collection dates between 1990 and 2012. These were used for molecular characterization. We estimated the maximum clade credibility (MCC) tree using a Bayesian analysis of core protein (C) gene sequences with ~660 bp of hepatitis B virus strains. The results are shown in Figure 1. The phylogeny showed that (1) (A/B/D) genotypes were clustered together. The blue color depicts genotype A (n = 55), and pink and green color display, genotypes D (n = 54) and B (n = 44), respectively; (2) genotypes A and D appeared to have a closer evolutional relationship than genotype B. However, they branched laterally from genotype B at an earlier time point; (3) genotype D also has subtypes. The various genotypes of HBV have differences in terms of levels of replication, and expression of viral markers. In addition, natural variations of HBV may occur in the process of the infection in the host. The accumulation of these variations can result in large changes in genomic nucleotide sequences of HBV, and eventually in the appearance of new genotypes.

### Evolutionary rate, TMRCA of each HBV genotype we collected

**Figure 2:**
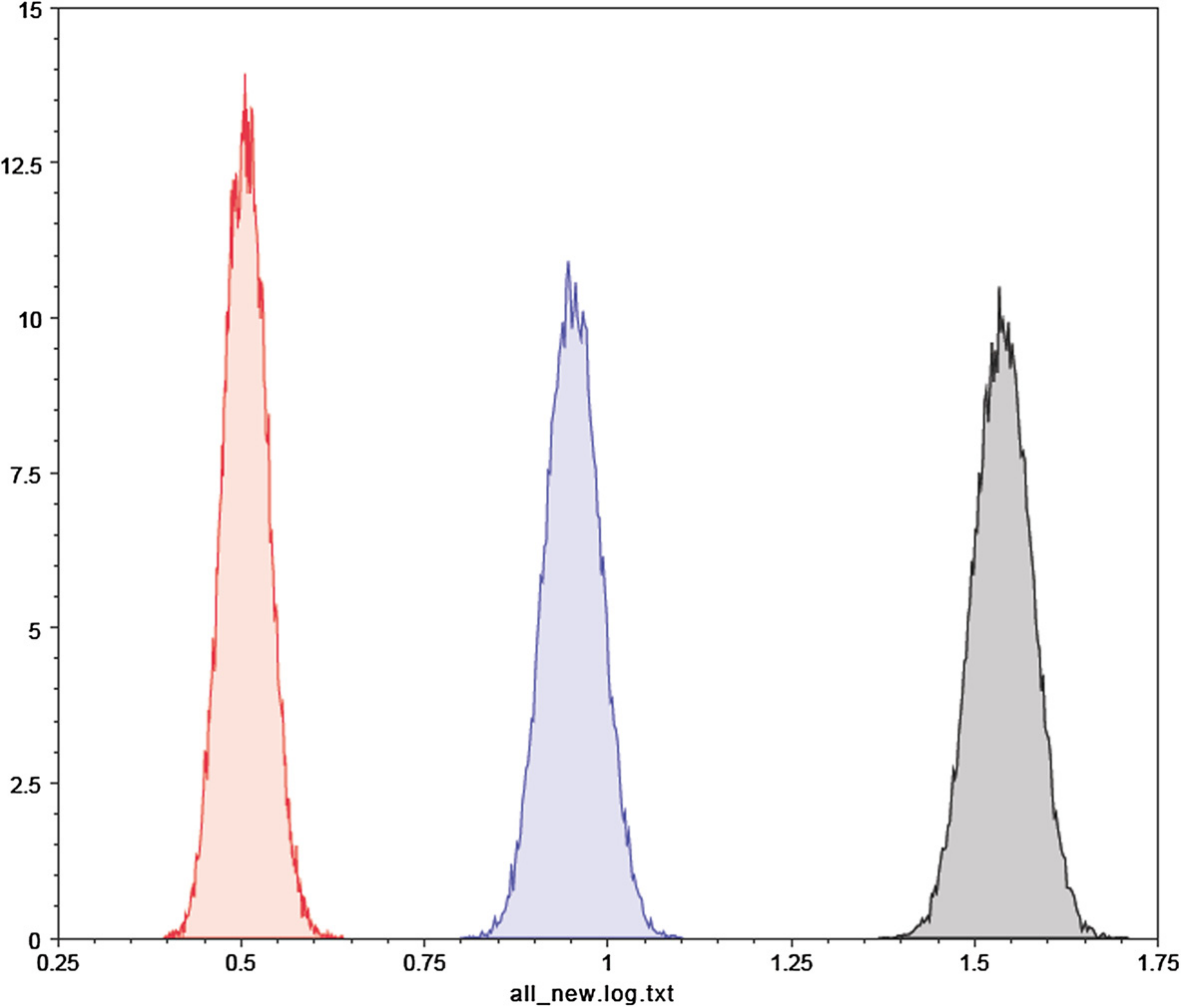
Marginal density of relative substitution rate densities for all three codon positions of the HBcAg gene.

**Figure 3:**
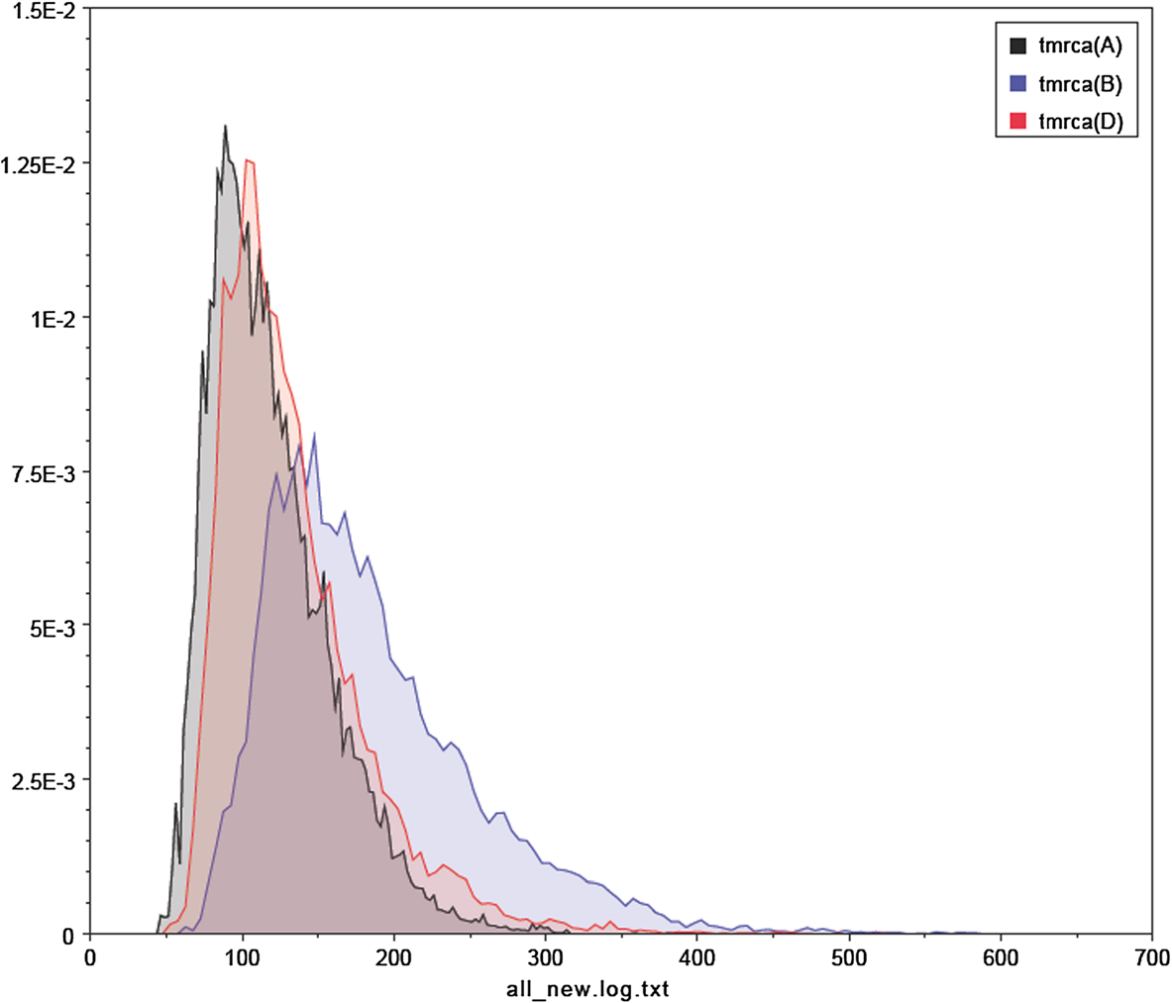
Marginal density of TMRCA for various HBV genotypes based on the HBcAg gene using an models of exponential population growth and a relaxed molecular clock, which were prepared by BEAST v1.6.1.

To understand the evolution of HBV, we estimated molecular clock phylogenies, evolution rates, and divergence times using the Bayesian MCMC method. All three codon positions of the HBcAg gene had different relative substitution rates (Table 1 and Figure 2). The mean values of the first, second, and third codon positions were 0.506, 1.539 and 0.953, respectively. Among these codon positions, the relative substitution rates of the second and the third codons were all greater than 1. Because the variance ratio was comparatively large, there was high variability and a high outbreak rate of HBV. At the same time, our analysis showed that the HBcAg gene evolutionary rate was estimated to be 1.127E-3 substitutions/site/year (Table 2 and Figure 3). The TMRCA of genotypes A, B and D of HBV were 118, 184 and 133, respectively so their corresponding emergence dates were calculated to be 1894, 1828 and 1879. Table 2 summarizes the dates of initial reports for each genotype included in our analysis. Someone used to calculate the evolutionary rates of HBV by the method of Bayesian algorithm and had found some specific evolutionary areas, these areas may have something to do with the high mutation rates of HBV [11]. The evolution of HBV was also be studied by the gene of HBsAg, its findings showed that HBV has always had high mutation rate in history [12]. Our study used different from these two papers. The first one used 108 HBV genomes. They indeed used more genomes in other genotypes except D. We actually download all HBV genomes and HBcAg region sequences and removed the genomes without region annotation. The second paper used HBsAg gene to do the analysis.

**Table 1:** Estimates of the relative substitution rates for the core gene of all three codon positions

**Summary statistic**	**CP1.mu**	**CP2.mu**	**CP3.mu**
Mean	0.506	1.539	0.953
95% HPD lower	0.448	1.458	0.881
95% HPD upper	0.567	1.618	1.030
Effective sample size (ESS)	12715	8705	5650

**Table 2:** Evolutionary characteristics of HBV genotypes based on the HBcAg gene using the uncorrelated log normal relaxed clock model produced by BEAST

**HBV genotype**	**Location, year reported**	**HBV TMRCA (years; 95% HPD)**	**Emergence time (year)**
		Substitution rate (CR)* 1.127 (0.925–1.329)	
TMRCA(A)	Japan,1988 [20]	118 (54–194)	1894
TMRCA (B)	Japan,1988 [20]	184 (78–323)	1828
TMRCA (D)	Japan,1988 [20]	133 (65–230)	1879

*Substitution rate are expressed as 10^-3^ substitutions per site per year.

### Dynamics of population growth

**Figure 4:**
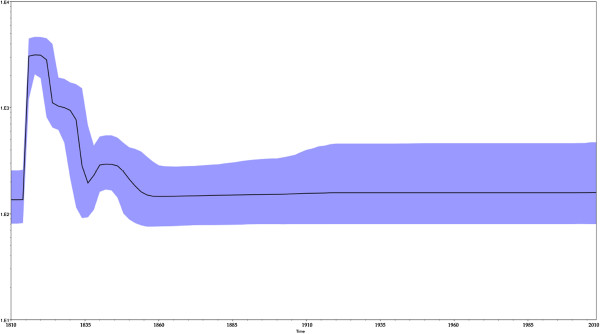
**The genetic diversity dynamics of HBV tree model, root height.** We can see that the plot for the HBcAg gene has a sharp rise in 1810 which for the relative genetic diversity.

Bayesian skyline plot analyses was used to investigate the dynamics of HBcAg gene genetic diversity over time (Figure 4), by which changes in genetic diversity can be observed as a function of time. There was a very sharp increase in relative genetic diversity (g) for the HBcAg gene in the first 10 years from 1810. The same tendency was observed for genotypes A, B, D according the maximum clade credibility (MCC) tree as shown in Figure 1.

The current study shows that the mutation rates for genotype A/B are much higher than that for genotype D. The reason for this may be that the populations included in these studies were mainly European and American, in which genotype A is predominant. in contrast to the situation in Eastern Asia where B genotype is predominant. In Europe and America, hepatitis B immune globulin (HBIG) is routinely used to block maternal-neonatal transmission [21], prevent HBV re-infection after liver transplantation [22], as well as to prevent infection after known acute exposures [23]. The observed increase in mutation rates could have been due to the effects of antiviral treatment [24], inoculation of hepatitis B vaccine or hepatitis B immune globulin [25].

Because the various genotypes of HBV exhibit different pathogenic features, and responses to drug treatment, the study of HBV genotypes has important clinical implications. HBV genotypes (1) can be useful for studies on epidemiology and regional distribution, pathogenicity and genetic variations, as well as emergence of mutational strains in various populations; (2) can suggest routes of transmission. For example, genotype A has been associated with sexual contact, while genotype D has been associated with blood transmission [14]; (3) the effectiveness of vaccines, particularly with regard to the relationship between genotype and maternal-neonatal transmission; (4) the relationship between genotypes and antiviral efficacy.

## Conclusions

We predicted the dynamic phylogenetic trends, which indicate outbreak trends of HBV, and provide theoretical foundations for clinical prevention and treatment of HBV.

## Competing interests

The authors declare that they have no competing interests.

## Authors’ contributions

ZN and WGQ conceived the study and designed the experiments. XGY and WCG analyzed the data and wrote the manuscript. GYQ and ZC contributed in data collection. All authors read and approved the final manuscript.
